# The Rat Sarcoma Virus (RAS) Family of Proteins in Sarcomas

**DOI:** 10.7759/cureus.57082

**Published:** 2024-03-27

**Authors:** Beytullah Unat

**Affiliations:** 1 Orthopedics and Traumatology, Gaziantep City Hospital, Gaziantep, TUR

**Keywords:** pulmonary fibrosis, non-coding rna, fibrosis, α-sma, acta2-as1

## Abstract

The rat sarcoma virus (RAS) protein family plays a crucial role in facilitating communication both within and between cells, thereby governing fundamental cellular processes such as growth, survival, and differentiation. The RAS family comprises four members of small GTPases, namely Harvey RAS (H-RAS), Kirsten RAS (K-RAS, two splice variants, 4A and 4B), and Neuroblastoma RAS (N-RAS), and these are encoded by three cellular RAS genes. Mutations in these genes play a significant role in cancer development and progression. Accordingly, here we review and discuss currently available literature about the fate and function of the RAS family of proteins in sarcomas.

## Introduction and background

Sarcomas, a hypotype of solid tumors originating from mesenchymal cells, exhibit a higher prevalence during childhood. Sarcomas constitute approximately 1% of all adult solid cancers, whereas they make up nearly 21% of childhood cancers [1, 2]. Sarcomas differ from other solid tumors in that they not only represent more than one malignancy but also have histologically different subgroups. However, they are grouped into two large subgroups, bone sarcomas and soft tissue sarcomas, according to their anatomical location. Soft tissue sarcomas are often found in muscles, joints, nerves, blood vessels, deep skin tissues, and fat. Bone sarcomas, on the other hand, are commonly found throughout all the bones of the body as well as in the cartilage [2, 3]. Commonly observed types of soft tissue sarcomas include fibrosarcoma, rhabdomyosarcoma, leiomyosarcoma, synovial sarcoma, liposarcoma, undifferentiated pleomorphic sarcoma, and gastrointestinal stromal tumors. Bone sarcomas, on the other hand, can be classified as Ewing's sarcoma, osteosarcoma, and chondrosarcoma [3, 4]. The existence of such various subtypes of sarcomas and their differences in location indicate the complexity of their molecular mechanisms. The risk of sarcoma has been shown to be increased in individuals with several known genetic syndromes. Examples include neurofibromatosis, retinoblastoma, and Li-Fraumeni syndromes [5]. The etiology of the majority of sarcomas is unknown; in addition to genetic risk factors, carcinogens, viruses, and ionizing radiation are shown to be environmental risk factors [5, 6].

Rat sarcoma virus (RAS) consists of GTP-binding proteins with low molecular weight belonging to the GTPase superfamily, which convert extracellular signals into various cellular functions. RAS is involved in various important cellular signal transduction pathways that mainly regulate cell growth, differentiation, cell adhesion and migration, and survival [7, 8]. The RAS family has been well studied due to their critical roles in tumorigenesis and progression. It has been shown that genes in the RAS signaling pathway are frequently mutated in many types of solid tumors, including breast, lung, colon, ovarian, and hematological malignancies [9]. Accordingly, in this comprehensive review, the significance of the RAS signaling pathway, its mutation status, and its effects on the pathogenesis of sarcoma types have been discussed.

## Review

RAS signaling pathway

The RAS superfamily consists of hundreds of different members, and RAS oncogene proteins are divided into five main subgroups: Ras, Rho, Rab, Ran, and Arf, according to their sequence and functional similarities [10]. The RAS molecule is a low molecular weight membrane-bound protein and functions as molecular switches regulated by Ras superfamily GTPases [11]. RAS superfamily proteins exhibit high-affinity binding for GDP and GTP and have low intrinsic GTP hydrolysis and GDP/GTP exchange activities. Canonically, small GTPases exist in the active GTP-bound or inactive GDP-bound state; their transformation depends on GTPase-activating protein (GAP) and guanine nucleotide exchange factors (GEFs). GEFs promote the formation of the active, GTP-bound form, while GAPs accelerate endogenous GTPase activity to promote the formation of the inactive, GDP-bound form [10, 12, 13]. RAS GTPases, the molecular switches of the cell, regulate signaling relays from various receptors. The canonical RAS gene encodes four protein isoforms: Harvey RAS (H-RAS), Kirsten RAS (K-RAS), and Neuroblastoma RAS (N-RAS) [14].

RAS activates the RAF, RAF activates MEK, and MEK sends signals to extracellular signal-regulated kinase (ERK) to activate growth-promoting gene expression. For this activation, first, extracellular signaling molecules activate RAS by binding to a receptor tyrosine kinase (RTK), and active RAS recruits RAF to the plasma membrane. When RAF is activated at the cell membrane, it phosphorylates downstream MEK, which in turn phosphorylates ERK [15, 16]. RAS mutations are frequently encountered in cancer. Among other types of tumors, the frequency of K-RAS mutation is 7% in soft tissue tumors and 1% in bone tumors; N-RAS mutation frequency is 4% in soft tissue tumors; H-RAS mutation has been shown to occur at a rate of 5% in soft tissue tumors and 2% in bone tumors [16].

Soft tissue sarcomas

Soft tissue sarcomas are rare, heterogeneous malignant soft tissue tumors that develop from mesodermal tissue, primarily connective, muscular, fatty, neural, vascular, and lymphatic tissues. [17]. The extremities are the most common site for soft tissue sarcoma; therefore, orthopedic surgeons must be knowledgeable about these types of tumors in order to evaluate a patient with a soft tissue mass correctly. Appropriate diagnosis and management of these patients are necessary for optimal outcomes. Despite their histological variety, soft tissue sarcomas share biological characteristics in common based on size, location, and histological grade [17, 18]. Soft tissue sarcomas grow centripetally and are surrounded by a pseudocapsule. Sarcomas, unlike invasive tumors, causes compression of the surrounding tissues and neurovascular structures as they expand. Therefore, recurrence rates are higher after resection [19]. The average five-year survival rate of soft tissue sarcomas has been determined to be 65%. However, this rate varies significantly depending on whether it is local or metastatic [17-19]. The relationship between RAS signaling and soft tissue sarcomas is thoroughly addressed in the subsequent subsections.

Rhabdomyosarcoma

Rhabdomyosarcomas constitute the largest category of soft tissue sarcomas in children and adolescents. According to the defined clinicopathological features and genetic abnormalities, rhabdomyosarcomas are divided into embryonal, alveolar, spindle cell/sclerosing, and pleomorphic subtypes [20, 21]. Embryonal rhabdomyosarcomas (eRSM) are the most common subtype of rhabdomyosarcoma. This subtype is most common in children younger than five years of age, and the overall survival of patients in the localized stage at five years averages 70% [22]. Copy number variations and RAS pathway mutations are frequently seen in eRSM. Stratton et al. showed that 35% of patients with eRSM had N-RAS or K-RAS mutations [23]. In addition, it has been reported that RAS pathway components FGFR4, RAS, NF1, and PIK3CA are activated by mutation [24]. In the study conducted by Shukla et al., RAS mutations were found in 11.7% of eRSM patients and 3.5% of alveolar RMS (aRMS) samples. In addition, mutations in the BRAF gene, an important component of the RAS signaling pathway, were detected in 1.7% of eRMS samples. It has also been shown that some of these patients have the activating BRAFV600E mutation [25]. Martinelli et al. reported that six of 31 primary eRMS samples had an N-RAS mutation, and one had an H-RAS mutation, but no K-RAS mutation was observed in these patients [26]. In another study conducted on eRSM patients, it was determined that two patients had K-RAS K117N mutations, one had an H-RAS mutation, and seven had an N-RAS mutation [27]. An accumulating mass of evidence suggests that such activating mutations in the RAS pathway are significantly involved in the pathogenesis and progression of eRSM tumors. In addition, it has been shown that TP53 plays a role in RMS formation by contributing to RAS activation in mouse RMS models with RAS gain of function and TP53 loss of function [28]. Based on studies conducted in eRMS, it has been determined that N-EAS is mutated more frequently than others [25, 27]. Alveolar RMS (aRMS) is the most aggressive undifferentiated subtype of RMS. Specifically, aRMS is characterized by t(2;13)(q35;q14) translocation in 70% of cases and t(1;13)(p36;q14) translocation in a smaller proportion of cases. These translocations generate PAX3-FOXO1 and PAX7-FOXO1 fusion genes, which encode PAX3-FOXO1 and PAX7-FOXO1 fusion proteins known to play a role in cell survival and cell cycle in aRMS cells [29, 30]. RASGRF1, a GTP exchange factor, has been found to be increased in aRMS tissues and cells. Moreover, when RASGRF1 is silenced using shRNA, cell proliferation is inhibited. RASGRF1 also contributes to signaling from crucial receptors such as CXCR4, c-met, Igf-1R, and Ins-R, which play a vital role in the migration, metastasis, and growth of aRMS cells [31].

Synovial sarcoma

Synovial sarcoma is a rare and poorly differentiated soft tissue sarcoma that constitutes 10% of all soft tissue sarcomas [32]. Synovial sarcoma can occur at any age, but it is more commonly observed in individuals during adolescence and in adults under the age of 30. The incidence of the disease varies by age, and approximately 1000 patients are diagnosed with synovial sarcoma each year in the United States [33]. The most common genetic modification in synovial sarcoma is the t(X;18)(p11.2;q11.2) translocation between chromosomes X and 18. Following this translocation, several different SS18:SSX fusion proteins are expressed. Fusion resulting from translocation is an important diagnostic tool, as it is detected in more than 95% of patients [34]. In addition, epidermal growth factor receptor (EGFR) has been reported to be overexpressed in synovial sarcoma compared to other soft tissue cancers, and high EGFR expression has been associated with poor prognosis [35, 36]. Peng et al. showed that sorafenib suppressed growth and stimulated apoptosis in synovial sarcoma cells by suppressing the RAF/MEK/ERK signaling pathway, which is the downstream effector of EGFR [35]. On the other hand, Dobashi and colleagues reported in their study that although EGFR expression was detected in both malignant and benign tumors, EGFR activation and gene abnormalities were only in sarcomas [37]. High EGFR amplification in sarcomas has been shown to be associated with STAT-3 activation. STAT-3 activated in bone tumors such as Ewing's sarcoma has been reported to be a result of continuous activation of EGFR signaling [37, 38]. In a study conducted by Oda et al., it was reported that H-RAS mutation was present in a small number of synovial sarcoma cases, and this mutation was not associated with the clinicopathological characteristics of the patients [39]. According to Array comparative genomic hybridization (CGH) results, it has been reported that there is a loss of RAS association domain family 1A (RASSF1) in patients with synovial sarcoma and that RASSF1 loss is associated with low RASSF1A protein level [40, 41]. In addition, in the study by Numoto et al., it was determined that the RASSF1A methylation rate was higher in synovial sarcoma patients compared to other soft tissue sarcomas. Additionally, RASSF1A was shown to be downregulated due to promoter methylation [42]. Another study reported that RASSF1A promoter methylation may be a prognostic marker in soft tissue sarcomas [43].

Liposarcoma

Liposarcoma is a rare type of cancer but has a high rate of recurrence and metastasis. Liposarcomas are subdivided into well-differentiated liposarcomas, dedifferentiated liposarcomas, myxoid liposarcomas, and pleomorphic liposarcomas [44-46]. The best characteristic feature of well-differentiated and dedifferentiated liposarcomas is expressed as the presence of many ring and giant rod chromosomes. These chromosomes have been shown to be from the chromosomal region 12q13-15 [47, 48]. Almost 95% of myxoid liposarcoma patients have a t(12;16)(q13;p11) translocation, and as a result of this translocation, FUS-CHOP gene fusion occurs [49, 50]. Pleomorphic liposarcomas constitute a very small portion of all liposarcomas, and their mortality has been reported to be around 40% [51]. As with other liposarcomas, there are no detailed studies on the clear molecular genetics of pleomorphic liposarcomas. However, in the literature, losses have been observed at the chromosomal level, and genetic variations such as deletions containing some tumor suppressor genes have been shown [52, 53].

In a study conducted by Ishii et al. in 63 patients with liposarcoma, they reported that pmTOR and pMEK positivity were observed more frequently in dedifferentiated lesions compared to well-differentiated ones [54]. Additionally, the combined use of mTOR inhibitor RAD001 and MEK inhibitor PD0325901 has been shown to suppress cell proliferation in dedifferentiated liposarcoma cells [54]. Sakamato et al., in their study on dedifferentiated liposarcomas, showed that four out of 19 patients had an H-RAS mutation, and 15 well-differentiated liposarcoma patients had no H-RAS mutation [55].

Leiomyosarcoma

Leiomyosarcoma is a malignant soft tissue sarcoma composed of cells displaying distinct characteristics of the smooth muscle lineage. It is commonly seen in anatomical regions such as the uterus, deep extremities, blood vessels, retroperitoneum, and superficial dermis [56]. In a study conducted by Hill et al., it was shown that 14% of leiomyosarcoma patients had K-RAS mutation, and K-RAS mutation carriers had lower overall survival rates than others [57]. In addition, two major K-RAS variants (K-RAS 4A and K-RAS 4B) have been reported to be upregulated in malignant tissues of leiomyosarcoma patients [58]. It has been shown that hypermethylation of RASSF1A, which has an important role in the RAS pathway, is frequently seen in leiomyosarcoma and is associated with poor prognosis [43].

Bone sarcomas

Bone sarcomas are mesenchymal tumors that originate from bone and consist of very heterogeneous subtypes. Bone sarcoma formation can be explained by at least one oncogenic event and a sufficient microenvironment that leads to the emergence of cancer, its subsequent growth, and potential migration to distant organs. 

Osteosarcoma

Osteosarcoma is a bone tumor that is mostly seen in children and adolescents. Osteosarcoma exhibits aggressive biological behavior characterized by frequent vascular invasion, early metastasis, and a high rate of local recurrence. Osteosarcoma seen during adolescence is caused by linear bone growth, and tumors during this period are usually seen in bones where rapid growth occurs, such as the arms, legs, and shoulders. In addition, it has been reported that hereditary diseases such as Li-Fraumeni syndrome, retinoblastoma syndrome, and RECQ disorders are also involved in the etiology of osteosarcoma [59].

Na et al., in a study with 61 osteosarcoma patients, reported that RAF-1, pMEK1/2, and pERK1/2 were upregulated in tumor tissue, while these proteins were not expressed in normal bone tissue [60]. Furthermore, expression of RAF-1, pMEK1/2, and pERK1/2 was associated with the formation of distant metastases and poor overall survival. pMEK1/2 has been shown to be an independent prognostic biomarker for poor disease-free survival and survival [60]. Using bioinformatic analyses, Chen et al. showed that the RAS pathway may be a prognostic marker for osteosarcoma and that RAS-related genes are involved in molecular pathways related to tumor progression and immune infiltration [61]. In addition, it has been reported that macrophage migration inhibitory factor is upregulated in osteosarcoma tissues, and this protein triggers the RAS/MAPK pathway in a time- and dose-dependent manner and increases proliferation. Additionally, deletion of the macrophage migration inhibitory factor has been shown to suppress lung metastasis [62].

RASSF1A, which also exhibits tumor suppressor properties in soft tissue and bone tissue cancers, has been shown to be low expressed in osteosarcoma, and low expression is associated with a poor clinical course [63]. Additionally, overexpression of RASSF1A in osteosarcoma cell lines has been shown to control proliferation, cell migration, and invasion and to induce apoptosis through cell cycle arrest [63]. On the other hand, RRAS2, a small GTP-binding protein that is a member of the RAS superfamily, has been reported to be important in malignant transformation by playing an important role in regulating RAS signaling [64]. Moreover, RRAS2 was also reported to be upregulated in osteosarcoma, and its high expression is associated with poor prognosis. Additionally, RNAi-mediated suppression of RRAS2 has been shown to reduce cell proliferation and migration by suppressing the MEK/ERK signaling pathway [65]. 

Ewing's sarcoma

Ewing's sarcoma is a bone and soft tissue tumor frequently seen in children, adolescents, and teenagers. Treatment-resistant metastases are observed in the majority of patients, and the survival rate in metastatic and recurrent tumors is quite low [66, 67]. EWS-FLI1 gene fusion, which occurs as a result of t(11;22)(q12;q24) chromosomal translocation, has an important role in the diagnosis of Ewing's sarcoma [68].

Furthermore, RAS GTPase activating protein 1 (RACGAP1) was found to be upregulated in Ewing's sarcoma tissues and negatively correlated with the survival rates of patients [69]. In addition, it has been shown that the expression level of RASSF2 is significantly reduced in unmethylated patients compared to hypermethylated patients, and RASSF2 methylation may be a strong prognostic marker [70].

Chondrosarcoma

Chondrosarcomas are the second most common bone tumor, and the rate of metastasis, recurrence, and mortality after surgical resection varies. Conventional bone chondrosarcoma accounts for approximately 85% of all chondrosarcomas and can be classified as primary central and secondary peripheral chondrosarcomas according to their location in the bone. While most of the conventional chondrosarcomas develop according to their central location in the medullary space, the other part develops on the bone surface [71, 72]. Rare chondrosarcomas can be listed as dedifferentiated chondrosarcomas, mesenchymal chondrosarcomas, and clear cell chondrosarcomas [73]. Sakamoto and colleagues analyzed codon 12 and 13 H-RAS mutations using the PCR-RFLP method in 11 patients with conventional chondrosarcoma and nine patients with dedifferentiated chondrosarcoma. H-RAS mutation was detected in only two dedifferentiated chondrosarcoma patients [74].

## Conclusions

The RAS protein family serves as a pivotal component in cellular communication, influencing crucial aspects such as growth, survival, and differentiation. Consequently, it is unsurprising that this family of proteins contributes to the pathobiology of human sarcomas. The collective understanding of sarcoma pathogenesis holds promise for developing targeted therapeutic strategies to disrupt the aberrant signaling orchestrated by the RAS protein family in these cancer types.
